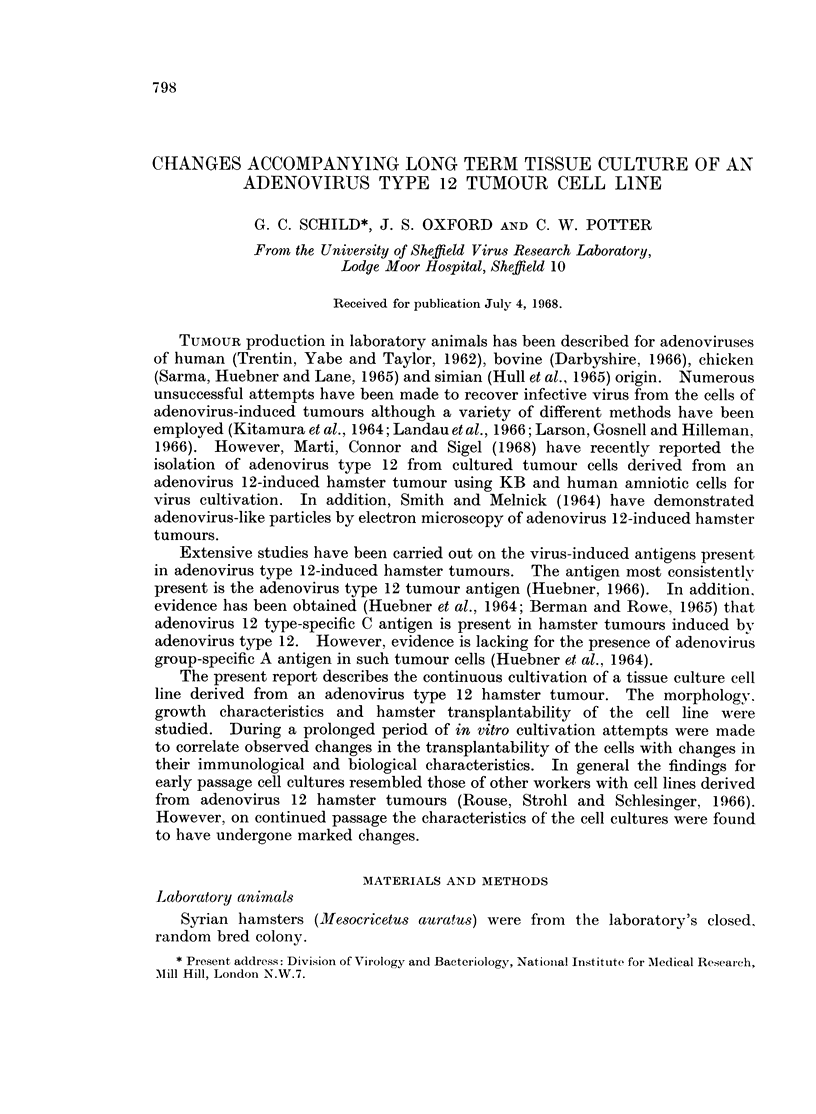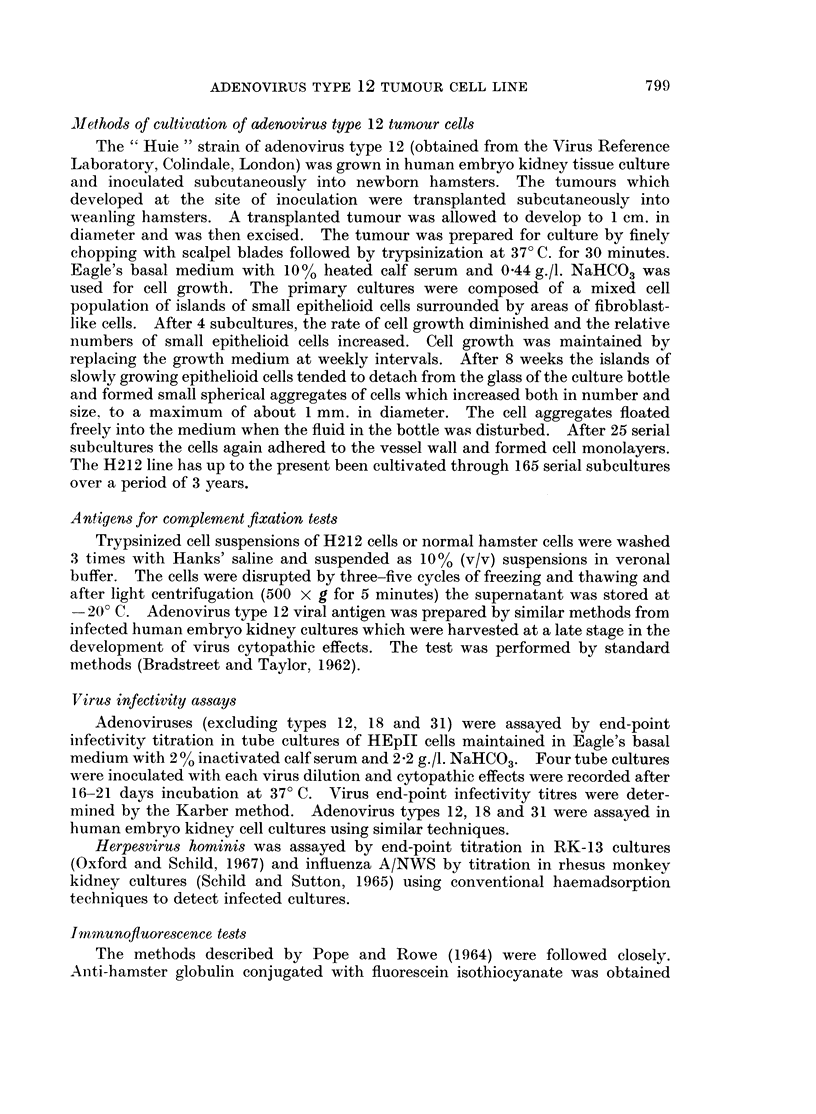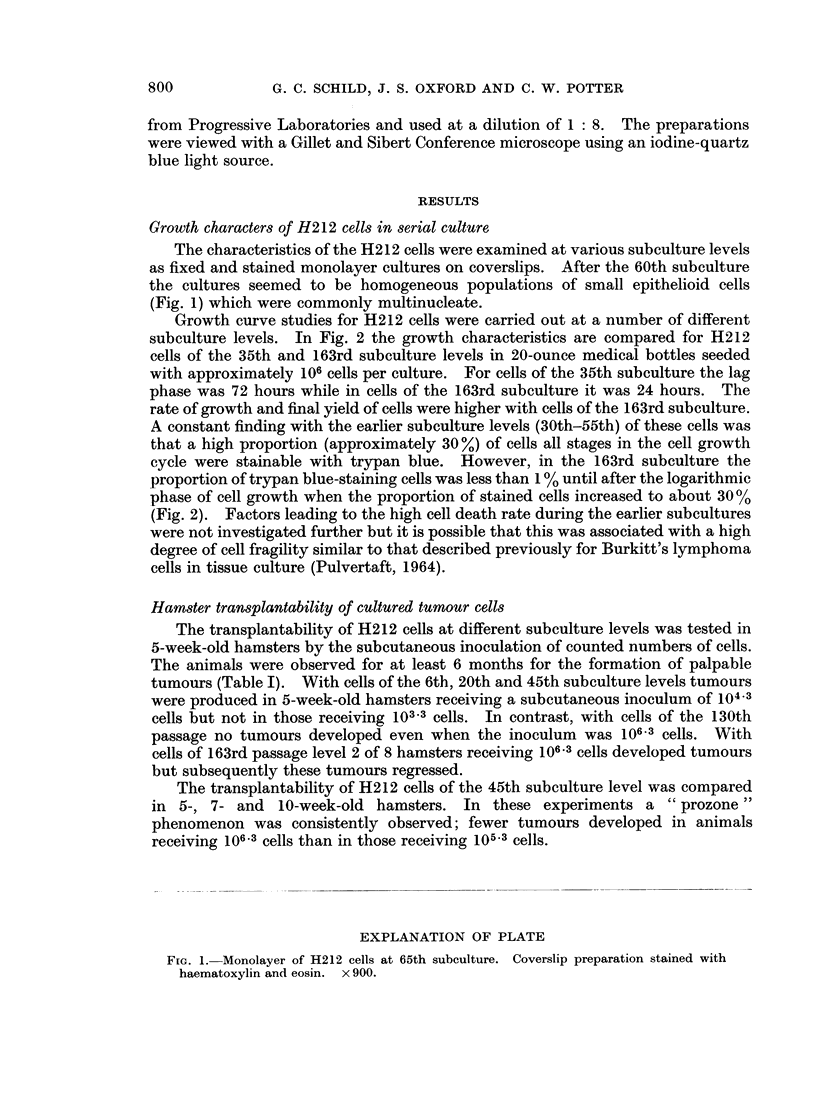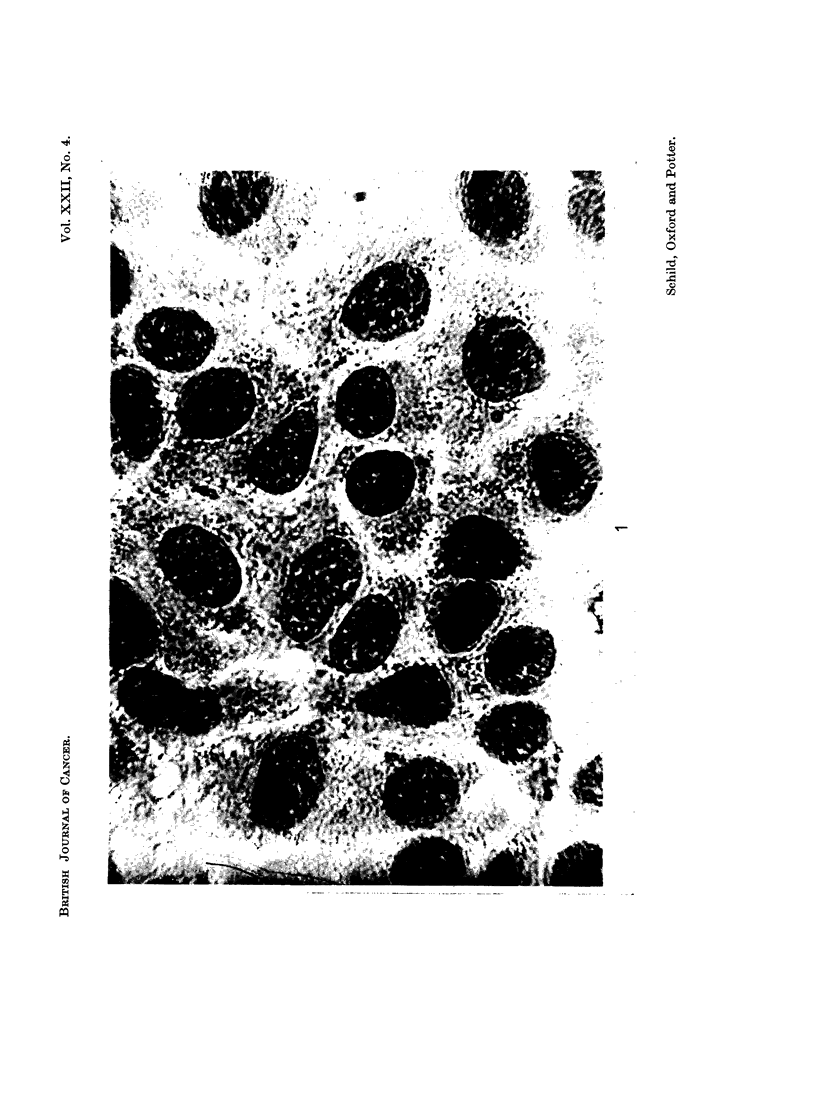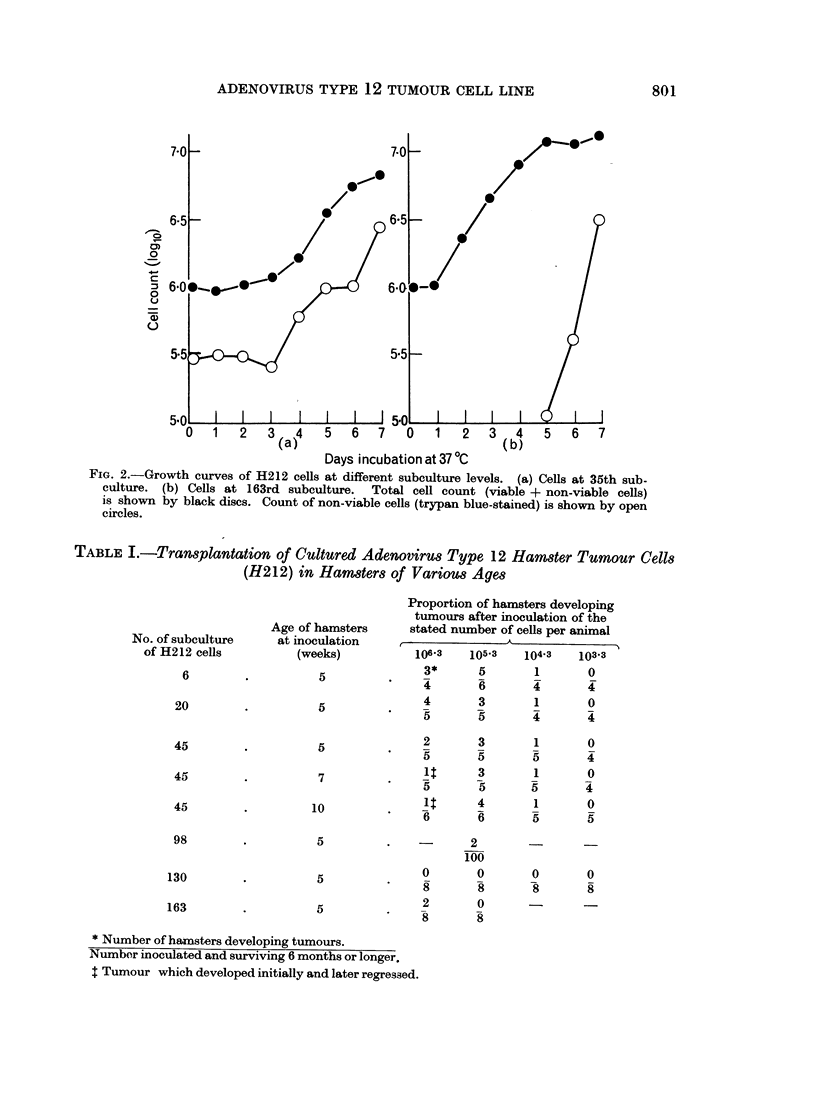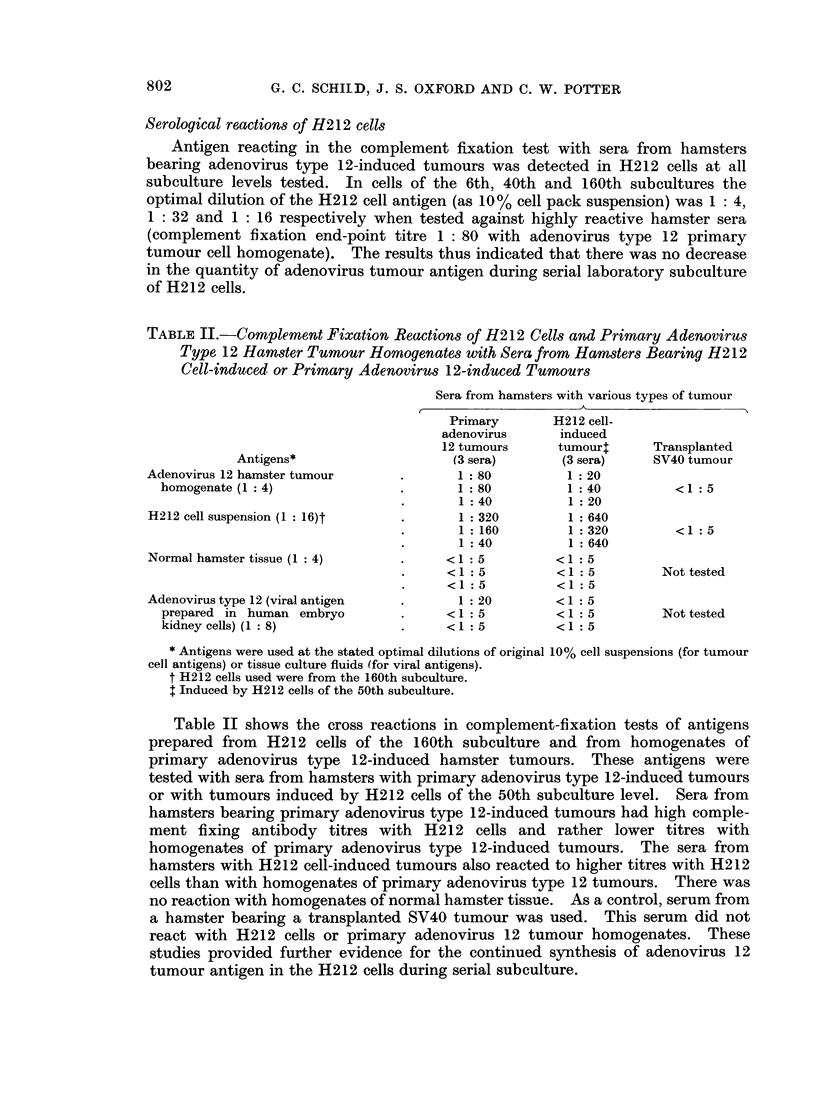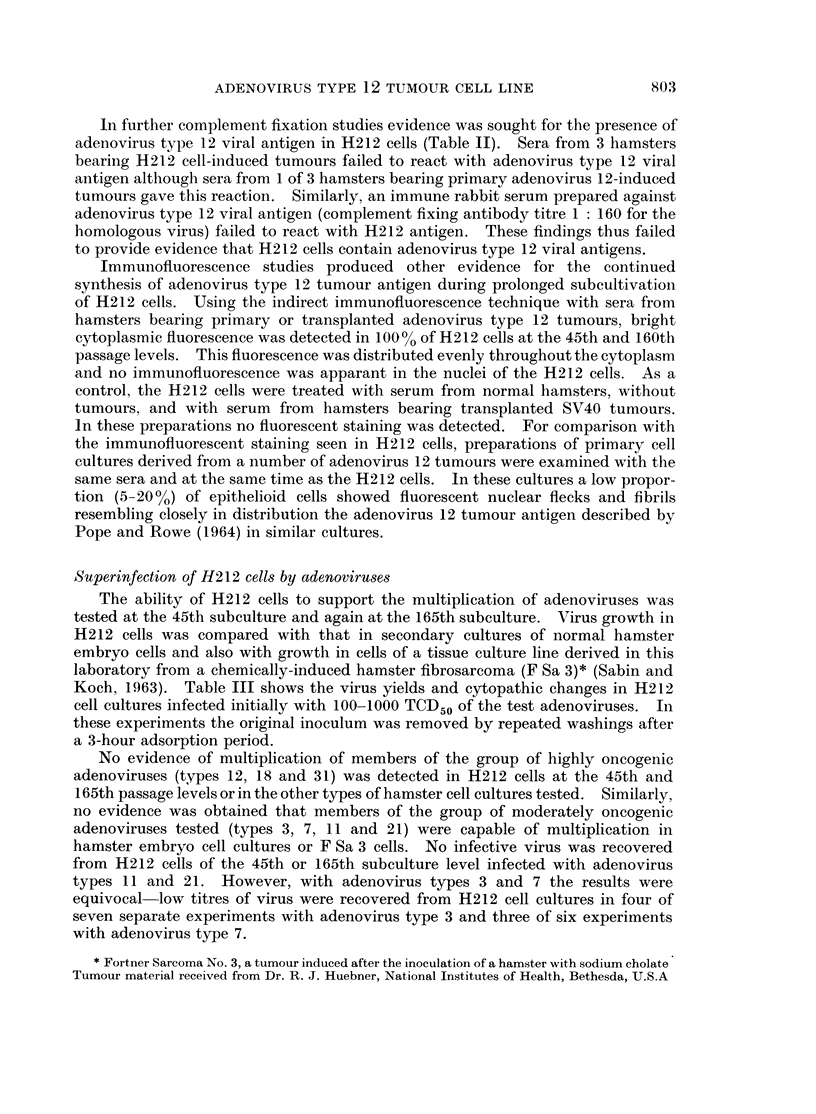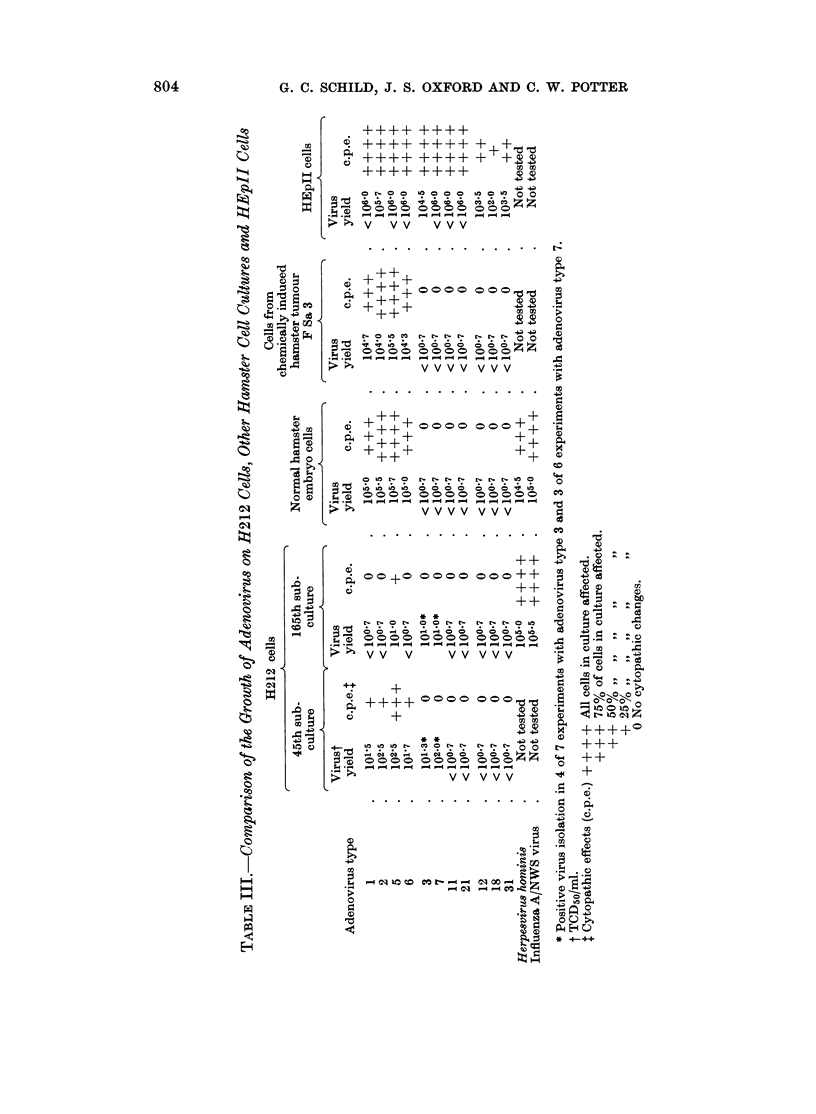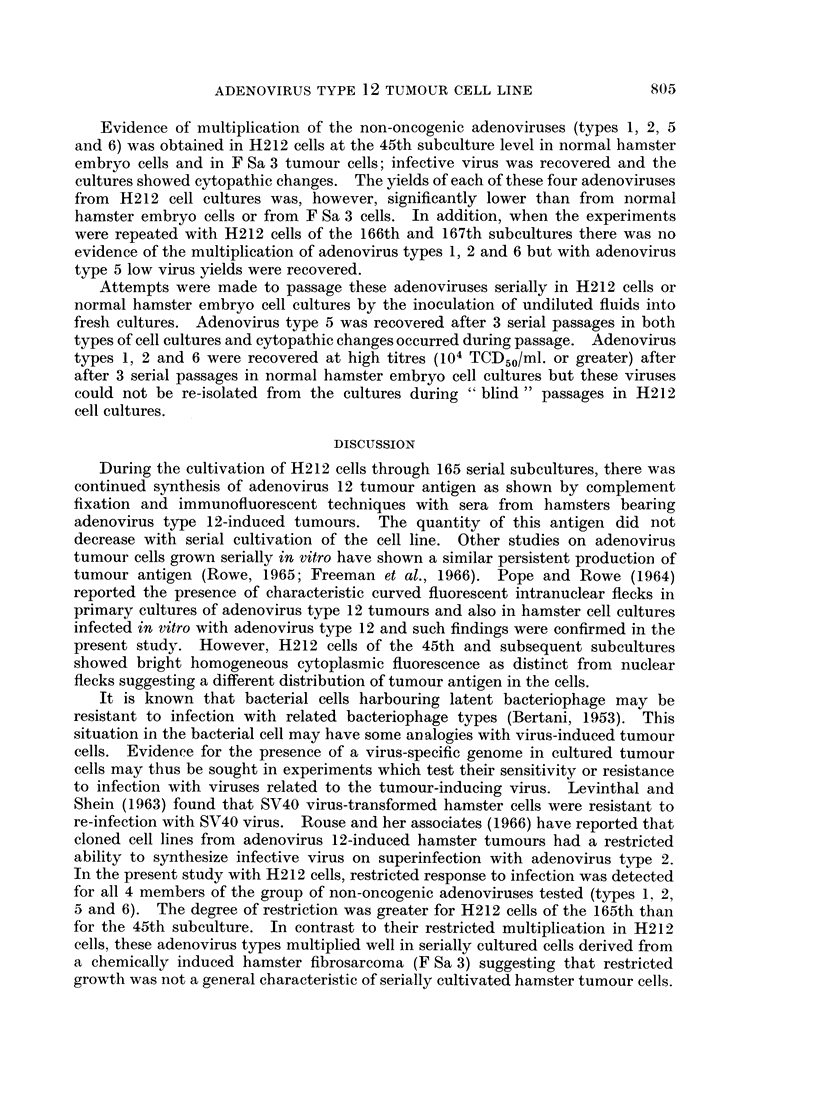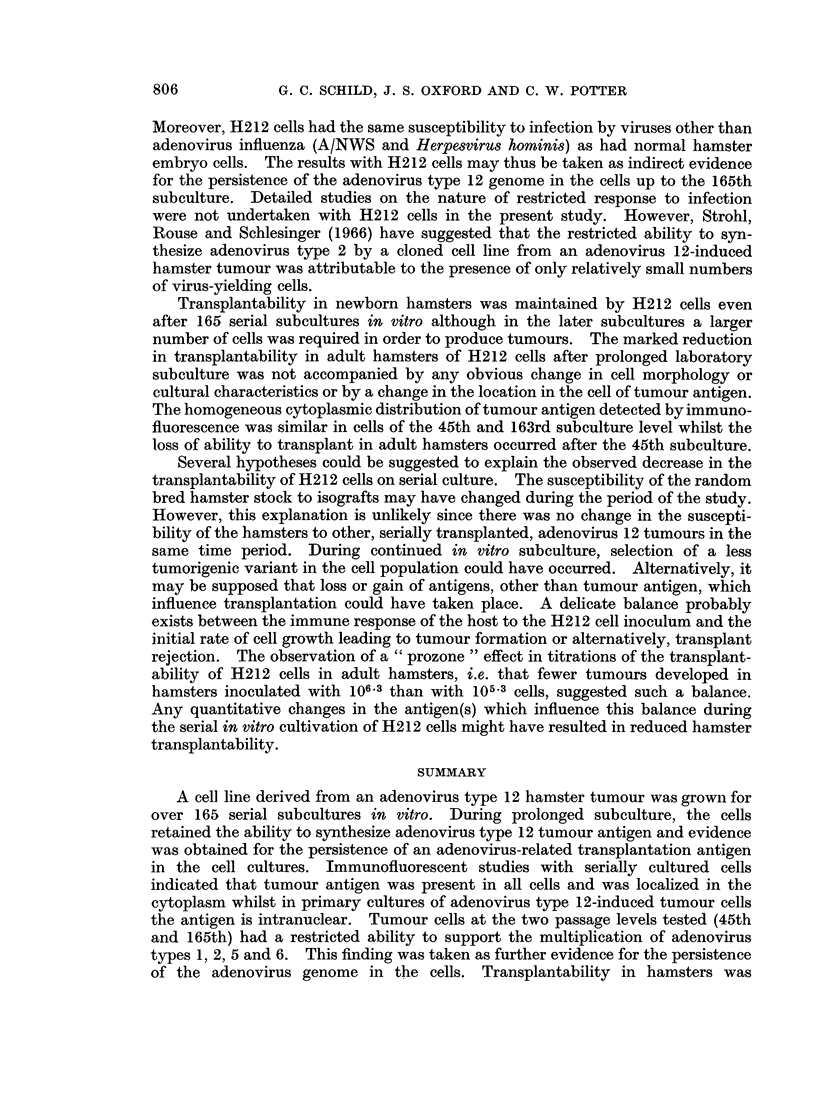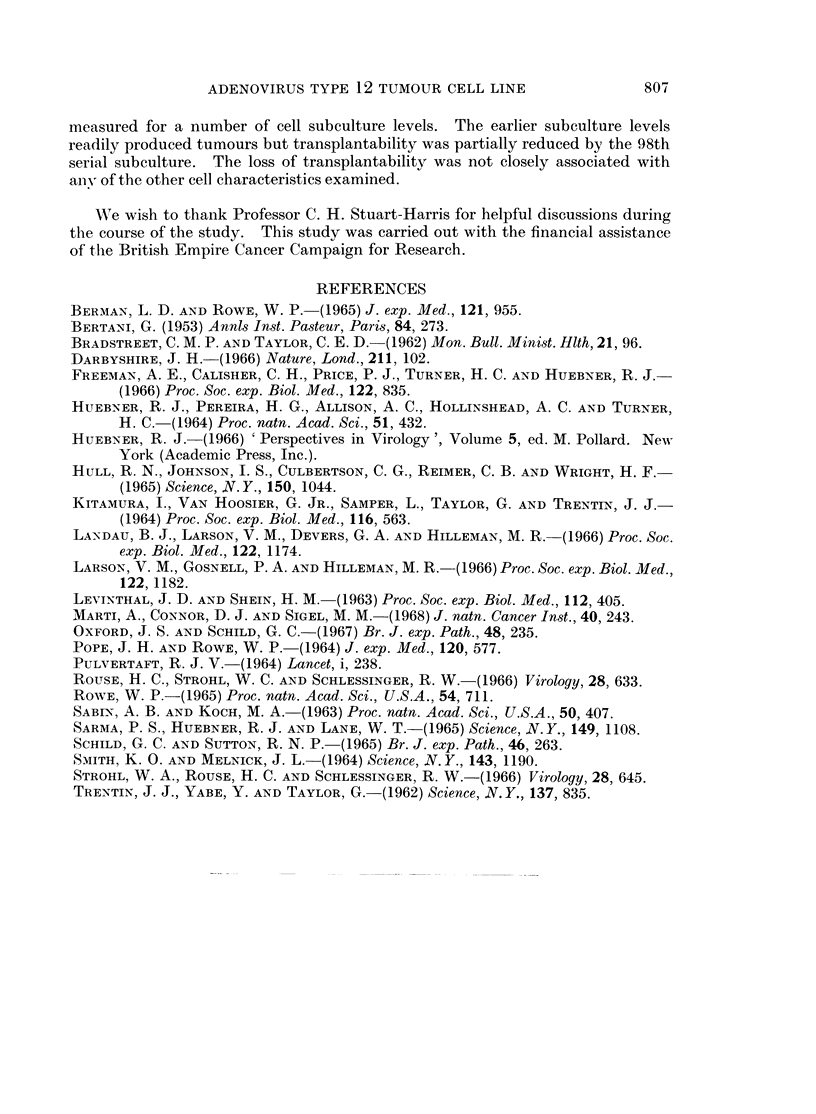# Changes accompanying long term tissue culture of an adenovirus type 12 tumour cell line.

**DOI:** 10.1038/bjc.1968.94

**Published:** 1968-12

**Authors:** G. C. Schild, J. S. Oxford, C. W. Potter

## Abstract

**Images:**


### CHANGES ACCOMPANYING LONG TERM TISSUE CULTURE OF AN ADENOVIRUS TYPE 12 TUMOUR CELL LINE

G. C. SCHILD*, J. S. OXFORD AND C. W. POTTER

*From the University of Sheffield Virus Research Laboratory, Lodge Moor Hospital, Sheffield* 10

Received for publication July 4, 1968.

TUMOUR production in laboratory animals has been described for adenoviruses of human (Trentin, Yabe and Taylor, 1962), bovine (Darbyshire, 1966), chicken (Sarma, Huebner and Lane, 1965) and simian (Hull *et al.*, 1965) origin. Numerous unsuccessful attempts have been made to recover infective virus from the cells of adenovirus-induced tumours although a variety of different methods have been employed (Kitamura *et al.*, 1964; Landau *et al.*, 1966; Larson, Gosnell and Hilleman, 1966). However, Marti, Connor and Sigel (1968) have recently reported the isolation of adenovirus type 12 from cultured tumour cells derived from an adenovirus 12-induced hamster tumour using KB and human amniotic cells for virus cultivation. In addition, Smith and Melnick (1964) have demonstrated adenovirus-like particles by electron microscopy of adenovirus 12-induced hamster tumours.

Extensive studies have been carried out on the virus-induced antigens present in adenovirus type 12-induced hamster tumours. The antigen most consistently present is the adenovirus type 12 tumour antigen (Huebner, 1966). In addition, evidence has been obtained (Huebner *et al.*, 1964; Berman and Rowe, 1965) that adenovirus 12 type-specific C antigen is present in hamster tumours induced by adenovirus type 12. However, evidence is lacking for the presence of adenovirus group-specific A antigen in such tumour cells (Huebner *et al.*, 1964).

The present report describes the continuous cultivation of a tissue culture cell line derived from an adenovirus type 12 hamster tumour. The morphology, growth characteristics and hamster transplantability of the cell line were studied. During a prolonged period of *in vitro* cultivation attempts were made to correlate observed changes in the transplantability of the cells with changes in their immunological and biological characteristics. In general the findings for early passage cell cultures resembled those of other workers with cell lines derived from adenovirus 12 hamster tumours (Rouse, Strohl and Schlesinger, 1966). However, on continued passage the characteristics of the cell cultures were found to have undergone marked changes.

### MATERIALS AND METHODS

### *Laboratory animals*

Syrian hamsters (*Mesocricetus auratus*) were from the laboratory's closed, random bred colony.

* Present address: Division of Virology and Bacteriology, National Institute for Medical Research, Mill Hill, London N.W.7.

*Methods of cultivation of adenovirus type* 12 *tumour cells*

The " Huie " strain of adenovirus type 12 (obtained from the Virus Reference Laboratory, Colindale, London) was grown in human embryo kidney tissue culture and inoculated subcutaneously into newborn hamsters. The tumours which developed at the site of inoculation were transplanted subcutaneously into weanling hamsters. A transplanted tumour was allowed to develop to 1 cm. in diameter and was then excised. The tumour was prepared for culture by finely chopping with scalpel blades followed by trypsinization at 37° C. for 30 minutes. Eagle's basal medium with 10% heated calf serum and 0·44 g./l. $NaHCO_3$ was used for cell growth. The primary cultures were composed of a mixed cell population of islands of small epithelioid cells surrounded by areas of fibroblast-like cells. After 4 subcultures, the rate of cell growth diminished and the relative numbers of small epithelioid cells increased. Cell growth was maintained by replacing the growth medium at weekly intervals. After 8 weeks the islands of slowly growing epithelioid cells tended to detach from the glass of the culture bottle and formed small spherical aggregates of cells which increased both in number and size, to a maximum of about 1 mm. in diameter. The cell aggregates floated freely into the medium when the fluid in the bottle was disturbed. After 25 serial subcultures the cells again adhered to the vessel wall and formed cell monolayers. The H212 line has up to the present been cultivated through 165 serial subcultures over a period of 3 years.

*Antigens for complement fixation tests*

Trypsinized cell suspensions of H212 cells or normal hamster cells were washed 3 times with Hanks' saline and suspended as 10% (v/v) suspensions in veronal buffer. The cells were disrupted by three–five cycles of freezing and thawing and after light centrifugation (500 $\times$ $g$ for 5 minutes) the supernatant was stored at −20° C. Adenovirus type 12 viral antigen was prepared by similar methods from infected human embryo kidney cultures which were harvested at a late stage in the development of virus cytopathic effects. The test was performed by standard methods (Bradstreet and Taylor, 1962).

*Virus infectivity assays*

Adenoviruses (excluding types 12, 18 and 31) were assayed by end-point infectivity titration in tube cultures of HEpII cells maintained in Eagle's basal medium with 2% inactivated calf serum and 2·2 g./l. $NaHCO_3$. Four tube cultures were inoculated with each virus dilution and cytopathic effects were recorded after 16–21 days incubation at 37° C. Virus end-point infectivity titres were determined by the Karber method. Adenovirus types 12, 18 and 31 were assayed in human embryo kidney cell cultures using similar techniques.

*Herpesvirus hominis* was assayed by end-point titration in RK-13 cultures (Oxford and Schild, 1967) and influenza A/NWS by titration in rhesus monkey kidney cultures (Schild and Sutton, 1965) using conventional haemadsorption techniques to detect infected cultures.

*Immunofluorescence tests*

The methods described by Pope and Rowe (1964) were followed closely. Anti-hamster globulin conjugated with fluorescein isothiocyanate was obtained from Progressive Laboratories and used at a dilution of 1 : 8. The preparations were viewed with a Gillet and Sibert Conference microscope using an iodine-quartz blue light source.

### RESULTS

### *Growth characters of H212 cells in serial culture*

The characteristics of the H212 cells were examined at various subculture levels as fixed and stained monolayer cultures on coverslips. After the 60th subculture the cultures seemed to be homogeneous populations of small epithelioid cells (Fig. 1) which were commonly multinucleate.

Growth curve studies for H212 cells were carried out at a number of different subculture levels. In Fig. 2 the growth characteristics are compared for H212 cells of the 35th and 163rd subculture levels in 20-ounce medical bottles seeded with approximately $10^6$ cells per culture. For cells of the 35th subculture the lag phase was 72 hours while in cells of the 163rd subculture it was 24 hours. The rate of growth and final yield of cells were higher with cells of the 163rd subculture. A constant finding with the earlier subculture levels (30th–55th) of these cells was that a high proportion (approximately 30%) of cells all stages in the cell growth cycle were stainable with trypan blue. However, in the 163rd subculture the proportion of trypan blue-staining cells was less than 1% until after the logarithmic phase of cell growth when the proportion of stained cells increased to about 30% (Fig. 2). Factors leading to the high cell death rate during the earlier subcultures were not investigated further but it is possible that this was associated with a high degree of cell fragility similar to that described previously for Burkitt's lymphoma cells in tissue culture (Pulvertaft, 1964).

### *Hamster transplantability of cultured tumour cells*

The transplantability of H212 cells at different subculture levels was tested in 5-week-old hamsters by the subcutaneous inoculation of counted numbers of cells. The animals were observed for at least 6 months for the formation of palpable tumours (Table I). With cells of the 6th, 20th and 45th subculture levels tumours were produced in 5-week-old hamsters receiving a subcutaneous inoculum of $10^{4\cdot3}$ cells but not in those receiving $10^{3\cdot3}$ cells. In contrast, with cells of the 130th passage no tumours developed even when the inoculum was $10^{6\cdot3}$ cells. With cells of 163rd passage level 2 of 8 hamsters receiving $10^{6\cdot3}$ cells developed tumours but subsequently these tumours regressed.

The transplantability of H212 cells of the 45th subculture level was compared in 5-, 7- and 10-week-old hamsters. In these experiments a "prozone" phenomenon was consistently observed; fewer tumours developed in animals receiving $10^{6\cdot3}$ cells than in those receiving $10^{5\cdot3}$ cells.

---

EXPLANATION OF PLATE

FIG. 1.—Monolayer of H212 cells at 65th subculture. Coverslip preparation stained with haematoxylin and eosin. ×900.

1

Schild, Oxford and Potter.

FIG. 2.—Growth curves of H212 cells at different subculture levels. (a) Cells at 35th subculture. (b) Cells at 163rd subculture. Total cell count (viable + non-viable cells) is shown by black discs. Count of non-viable cells (trypan blue-stained) is shown by open circles.

TABLE I.—*Transplantation of Cultured Adenovirus Type* 12 *Hamster Tumour Cells* (*H*212) *in Hamsters of Various Ages*

| No. of subculture of H212 cells | Age of hamsters at inoculation (weeks) | Proportion of hamsters developing tumours after inoculation of the stated number of cells per animal | | | |
|---|---|---|---|---|---|
| | | $10^{6\cdot3}$ | $10^{5\cdot3}$ | $10^{4\cdot3}$ | $10^{3\cdot3}$ |
| 6 | 5 | 3*/4 | 5/6 | 1/4 | 0/4 |
| 20 | 5 | 4/5 | 3/5 | 1/4 | 0/4 |
| 45 | 5 | 2/5 | 3/5 | 1/5 | 0/4 |
| 45 | 7 | 1‡/5 | 3/5 | 1/5 | 0/4 |
| 45 | 10 | 1‡/6 | 4/6 | 1/5 | 0/5 |
| 98 | 5 | — | 2/100 | — | — |
| 130 | 5 | 0/8 | 0/8 | 0/8 | 0/8 |
| 163 | 5 | 2/8 | 0/8 | — | — |

* Number of hamsters developing tumours / Number inoculated and surviving 6 months or longer.

‡ Tumour which developed initially and later regressed.

*Serological reactions of H212 cells*

Antigen reacting in the complement fixation test with sera from hamsters bearing adenovirus type 12-induced tumours was detected in H212 cells at all subculture levels tested. In cells of the 6th, 40th and 160th subcultures the optimal dilution of the H212 cell antigen (as 10% cell pack suspension) was 1 : 4, 1 : 32 and 1 : 16 respectively when tested against highly reactive hamster sera (complement fixation end-point titre 1 : 80 with adenovirus type 12 primary tumour cell homogenate). The results thus indicated that there was no decrease in the quantity of adenovirus tumour antigen during serial laboratory subculture of H212 cells.

TABLE II.—*Complement Fixation Reactions of H212 Cells and Primary Adenovirus Type 12 Hamster Tumour Homogenates with Sera from Hamsters Bearing H212 Cell-induced or Primary Adenovirus 12-induced Tumours*

| | Sera from hamsters with various types of tumour | | |
|---|---|---|---|
| Antigens* | Primary adenovirus 12 tumours (3 sera) | H212 cell-induced tumour‡ (3 sera) | Transplanted SV40 tumour |
| Adenovirus 12 hamster tumour homogenate (1 : 4) | 1 : 80 | 1 : 20 | |
| | 1 : 80 | 1 : 40 | <1 : 5 |
| | 1 : 40 | 1 : 20 | |
| H212 cell suspension (1 : 16)† | 1 : 320 | 1 : 640 | |
| | 1 : 160 | 1 : 320 | <1 : 5 |
| | 1 : 40 | 1 : 640 | |
| Normal hamster tissue (1 : 4) | <1 : 5 | <1 : 5 | |
| | <1 : 5 | <1 : 5 | Not tested |
| | <1 : 5 | <1 : 5 | |
| Adenovirus type 12 (viral antigen prepared in human embryo kidney cells) (1 : 8) | 1 : 20 | <1 : 5 | |
| | <1 : 5 | <1 : 5 | Not tested |
| | <1 : 5 | <1 : 5 | |

* Antigens were used at the stated optimal dilutions of original 10% cell suspensions (for tumour cell antigens) or tissue culture fluids (for viral antigens).
† H212 cells used were from the 160th subculture.
‡ Induced by H212 cells of the 50th subculture.

Table II shows the cross reactions in complement-fixation tests of antigens prepared from H212 cells of the 160th subculture and from homogenates of primary adenovirus type 12-induced hamster tumours. These antigens were tested with sera from hamsters with primary adenovirus type 12-induced tumours or with tumours induced by H212 cells of the 50th subculture level. Sera from hamsters bearing primary adenovirus type 12-induced tumours had high complement fixing antibody titres with H212 cells and rather lower titres with homogenates of primary adenovirus type 12-induced tumours. The sera from hamsters with H212 cell-induced tumours also reacted to higher titres with H212 cells than with homogenates of primary adenovirus type 12 tumours. There was no reaction with homogenates of normal hamster tissue. As a control, serum from a hamster bearing a transplanted SV40 tumour was used. This serum did not react with H212 cells or primary adenovirus 12 tumour homogenates. These studies provided further evidence for the continued synthesis of adenovirus 12 tumour antigen in the H212 cells during serial subculture.

In further complement fixation studies evidence was sought for the presence of adenovirus type 12 viral antigen in H212 cells (Table II). Sera from 3 hamsters bearing H212 cell-induced tumours failed to react with adenovirus type 12 viral antigen although sera from 1 of 3 hamsters bearing primary adenovirus 12-induced tumours gave this reaction. Similarly, an immune rabbit serum prepared against adenovirus type 12 viral antigen (complement fixing antibody titre 1 : 160 for the homologous virus) failed to react with H212 antigen. These findings thus failed to provide evidence that H212 cells contain adenovirus type 12 viral antigens.

Immunofluorescence studies produced other evidence for the continued synthesis of adenovirus type 12 tumour antigen during prolonged subcultivation of H212 cells. Using the indirect immunofluorescence technique with sera from hamsters bearing primary or transplanted adenovirus type 12 tumours, bright cytoplasmic fluorescence was detected in 100% of H212 cells at the 45th and 160th passage levels. This fluorescence was distributed evenly throughout the cytoplasm and no immunofluorescence was apparant in the nuclei of the H212 cells. As a control, the H212 cells were treated with serum from normal hamsters, without tumours, and with serum from hamsters bearing transplanted SV40 tumours. In these preparations no fluorescent staining was detected. For comparison with the immunofluorescent staining seen in H212 cells, preparations of primary cell cultures derived from a number of adenovirus 12 tumours were examined with the same sera and at the same time as the H212 cells. In these cultures a low proportion (5–20%) of epithelioid cells showed fluorescent nuclear flecks and fibrils resembling closely in distribution the adenovirus 12 tumour antigen described by Pope and Rowe (1964) in similar cultures.

*Superinfection of H212 cells by adenoviruses*

The ability of H212 cells to support the multiplication of adenoviruses was tested at the 45th subculture and again at the 165th subculture. Virus growth in H212 cells was compared with that in secondary cultures of normal hamster embryo cells and also with growth in cells of a tissue culture line derived in this laboratory from a chemically-induced hamster fibrosarcoma (F Sa 3)* (Sabin and Koch, 1963). Table III shows the virus yields and cytopathic changes in H212 cell cultures infected initially with 100–1000 $TCD_{50}$ of the test adenoviruses. In these experiments the original inoculum was removed by repeated washings after a 3-hour adsorption period.

No evidence of multiplication of members of the group of highly oncogenic adenoviruses (types 12, 18 and 31) was detected in H212 cells at the 45th and 165th passage levels or in the other types of hamster cell cultures tested. Similarly, no evidence was obtained that members of the group of moderately oncogenic adenoviruses tested (types 3, 7, 11 and 21) were capable of multiplication in hamster embryo cell cultures or F Sa 3 cells. No infective virus was recovered from H212 cells of the 45th or 165th subculture level infected with adenovirus types 11 and 21. However, with adenovirus types 3 and 7 the results were equivocal—low titres of virus were recovered from H212 cell cultures in four of seven separate experiments with adenovirus type 3 and three of six experiments with adenovirus type 7.

* Fortner Sarcoma No. 3, a tumour induced after the inoculation of a hamster with sodium cholate. Tumour material received from Dr. R. J. Huebner, National Institutes of Health, Bethesda, U.S.A

TABLE III.—*Comparison of the Growth of Adenovirus on H212 Cells, Other Hamster Cell Cultures and HEpII Cells*

| Adenovirus type | H212 cells: 45th subculture, Virus† yield | H212 cells: 45th subculture, c.p.e.‡ | H212 cells: 165th subculture, Virus yield | H212 cells: 165th subculture, c.p.e. | Normal hamster embryo cells, Virus yield | Normal hamster embryo cells, c.p.e. | Cells from chemically induced hamster tumour F Sa 3, Virus yield | Cells from chemically induced hamster tumour F Sa 3, c.p.e. | HEpII cells, Virus yield | HEpII cells, c.p.e. |
|---|---|---|---|---|---|---|---|---|---|---|
| 1 | $10^{1\cdot5}$ | + | $<10^{0\cdot7}$ | 0 | $10^{5\cdot0}$ | +++ | $10^{4\cdot7}$ | +++ | $<10^{6\cdot0}$ | ++++ |
| 2 | $10^{2\cdot5}$ | + | $<10^{0\cdot7}$ | 0 | $10^{5\cdot5}$ | ++++ | $10^{4\cdot0}$ | ++++ | $10^{5\cdot7}$ | ++++ |
| 5 | $10^{2\cdot5}$ | +++ | $10^{1\cdot0}$ | + | $10^{5\cdot7}$ | ++++ | $10^{5\cdot5}$ | ++++ | $<10^{6\cdot0}$ | ++++ |
| 6 | $10^{1\cdot7}$ | + | $<10^{0\cdot7}$ | 0 | $10^{5\cdot0}$ | +++ | $10^{4\cdot3}$ | +++ | $<10^{6\cdot0}$ | ++++ |
| 3 | $10^{1\cdot3}$* | 0 | $10^{1\cdot0}$* | 0 | $<10^{0\cdot7}$ | 0 | $<10^{0\cdot7}$ | 0 | $10^{4\cdot5}$ | ++++ |
| 7 | $10^{2\cdot0}$* | 0 | $10^{1\cdot0}$* | 0 | $<10^{0\cdot7}$ | 0 | $<10^{0\cdot7}$ | 0 | $<10^{6\cdot0}$ | ++++ |
| 11 | $<10^{0\cdot7}$ | 0 | $<10^{0\cdot7}$ | 0 | $<10^{0\cdot7}$ | 0 | $<10^{0\cdot7}$ | 0 | $<10^{6\cdot0}$ | ++++ |
| 21 | $<10^{0\cdot7}$ | 0 | $<10^{0\cdot7}$ | 0 | $<10^{0\cdot7}$ | 0 | $<10^{0\cdot7}$ | 0 | $<10^{6\cdot0}$ | ++++ |
| 12 | $<10^{0\cdot7}$ | 0 | $<10^{0\cdot7}$ | 0 | $<10^{0\cdot7}$ | 0 | $<10^{0\cdot7}$ | 0 | $10^{3\cdot5}$ | ++ |
| 18 | $<10^{0\cdot7}$ | 0 | $<10^{0\cdot7}$ | 0 | $<10^{0\cdot7}$ | 0 | $<10^{0\cdot7}$ | 0 | $10^{2\cdot0}$ | + |
| 31 | $<10^{0\cdot7}$ | 0 | $<10^{0\cdot7}$ | 0 | $<10^{0\cdot7}$ | 0 | $<10^{0\cdot7}$ | 0 | $10^{3\cdot5}$ | ++ |
| *Herpesvirus hominis* | Not tested | | $10^{5\cdot0}$ | ++++ | $10^{4\cdot5}$ | +++ | Not tested | | Not tested | |
| Influenza A/NWS virus | Not tested | | $10^{5\cdot5}$ | ++++ | $10^{5\cdot0}$ | ++++ | Not tested | | Not tested | |

* Positive virus isolation in 4 of 7 experiments with adenovirus type 3 and 3 of 6 experiments with adenovirus type 7.

† $TCD_{50}$/ml.

‡ Cytopathic effects (c.p.e.) ++++ All cells in culture affected.

+++ 75% of cells in culture affected.

++ 50% ,, ,, ,, ,, ,,

+ 25% ,, ,, ,, ,, ,,

0 No cytopathic changes.

Evidence of multiplication of the non-oncogenic adenoviruses (types 1, 2, 5 and 6) was obtained in H212 cells at the 45th subculture level in normal hamster embryo cells and in F Sa 3 tumour cells; infective virus was recovered and the cultures showed cytopathic changes. The yields of each of these four adenoviruses from H212 cell cultures was, however, significantly lower than from normal hamster embryo cells or from F Sa 3 cells. In addition, when the experiments were repeated with H212 cells of the 166th and 167th subcultures there was no evidence of the multiplication of adenovirus types 1, 2 and 6 but with adenovirus type 5 low virus yields were recovered.

Attempts were made to passage these adenoviruses serially in H212 cells or normal hamster embryo cell cultures by the inoculation of undiluted fluids into fresh cultures. Adenovirus type 5 was recovered after 3 serial passages in both types of cell cultures and cytopathic changes occurred during passage. Adenovirus types 1, 2 and 6 were recovered at high titres ($10^4$ $TCD_{50}$/ml. or greater) after after 3 serial passages in normal hamster embryo cell cultures but these viruses could not be re-isolated from the cultures during " blind " passages in H212 cell cultures.

### DISCUSSION

During the cultivation of H212 cells through 165 serial subcultures, there was continued synthesis of adenovirus 12 tumour antigen as shown by complement fixation and immunofluorescent techniques with sera from hamsters bearing adenovirus type 12-induced tumours. The quantity of this antigen did not decrease with serial cultivation of the cell line. Other studies on adenovirus tumour cells grown serially *in vitro* have shown a similar persistent production of tumour antigen (Rowe, 1965; Freeman *et al.*, 1966). Pope and Rowe (1964) reported the presence of characteristic curved fluorescent intranuclear flecks in primary cultures of adenovirus type 12 tumours and also in hamster cell cultures infected *in vitro* with adenovirus type 12 and such findings were confirmed in the present study. However, H212 cells of the 45th and subsequent subcultures showed bright homogeneous cytoplasmic fluorescence as distinct from nuclear flecks suggesting a different distribution of tumour antigen in the cells.

It is known that bacterial cells harbouring latent bacteriophage may be resistant to infection with related bacteriophage types (Bertani, 1953). This situation in the bacterial cell may have some analogies with virus-induced tumour cells. Evidence for the presence of a virus-specific genome in cultured tumour cells may thus be sought in experiments which test their sensitivity or resistance to infection with viruses related to the tumour-inducing virus. Levinthal and Shein (1963) found that SV40 virus-transformed hamster cells were resistant to re-infection with SV40 virus. Rouse and her associates (1966) have reported that cloned cell lines from adenovirus 12-induced hamster tumours had a restricted ability to synthesize infective virus on superinfection with adenovirus type 2. In the present study with H212 cells, restricted response to infection was detected for all 4 members of the group of non-oncogenic adenoviruses tested (types 1, 2, 5 and 6). The degree of restriction was greater for H212 cells of the 165th than for the 45th subculture. In contrast to their restricted multiplication in H212 cells, these adenovirus types multiplied well in serially cultured cells derived from a chemically induced hamster fibrosarcoma (F Sa 3) suggesting that restricted growth was not a general characteristic of serially cultivated hamster tumour cells.

Moreover, H212 cells had the same susceptibility to infection by viruses other than adenovirus influenza (A/NWS and *Herpesvirus hominis*) as had normal hamster embryo cells. The results with H212 cells may thus be taken as indirect evidence for the persistence of the adenovirus type 12 genome in the cells up to the 165th subculture. Detailed studies on the nature of restricted response to infection were not undertaken with H212 cells in the present study. However, Strohl, Rouse and Schlesinger (1966) have suggested that the restricted ability to synthesize adenovirus type 2 by a cloned cell line from an adenovirus 12-induced hamster tumour was attributable to the presence of only relatively small numbers of virus-yielding cells.

Transplantability in newborn hamsters was maintained by H212 cells even after 165 serial subcultures *in vitro* although in the later subcultures a larger number of cells was required in order to produce tumours. The marked reduction in transplantability in adult hamsters of H212 cells after prolonged laboratory subculture was not accompanied by any obvious change in cell morphology or cultural characteristics or by a change in the location in the cell of tumour antigen. The homogeneous cytoplasmic distribution of tumour antigen detected by immunofluorescence was similar in cells of the 45th and 163rd subculture level whilst the loss of ability to transplant in adult hamsters occurred after the 45th subculture.

Several hypotheses could be suggested to explain the observed decrease in the transplantability of H212 cells on serial culture. The susceptibility of the random bred hamster stock to isografts may have changed during the period of the study. However, this explanation is unlikely since there was no change in the susceptibility of the hamsters to other, serially transplanted, adenovirus 12 tumours in the same time period. During continued *in vitro* subculture, selection of a less tumorigenic variant in the cell population could have occurred. Alternatively, it may be supposed that loss or gain of antigens, other than tumour antigen, which influence transplantation could have taken place. A delicate balance probably exists between the immune response of the host to the H212 cell inoculum and the initial rate of cell growth leading to tumour formation or alternatively, transplant rejection. The observation of a " prozone " effect in titrations of the transplantability of H212 cells in adult hamsters, *i.e.* that fewer tumours developed in hamsters inoculated with $10^{6 \cdot 3}$ than with $10^{5 \cdot 3}$ cells, suggested such a balance. Any quantitative changes in the antigen(s) which influence this balance during the serial *in vitro* cultivation of H212 cells might have resulted in reduced hamster transplantability.

### SUMMARY

A cell line derived from an adenovirus type 12 hamster tumour was grown for over 165 serial subcultures *in vitro*. During prolonged subculture, the cells retained the ability to synthesize adenovirus type 12 tumour antigen and evidence was obtained for the persistence of an adenovirus-related transplantation antigen in the cell cultures. Immunofluorescent studies with serially cultured cells indicated that tumour antigen was present in all cells and was localized in the cytoplasm whilst in primary cultures of adenovirus type 12-induced tumour cells the antigen is intranuclear. Tumour cells at the two passage levels tested (45th and 165th) had a restricted ability to support the multiplication of adenovirus types 1, 2, 5 and 6. This finding was taken as further evidence for the persistence of the adenovirus genome in the cells. Transplantability in hamsters was measured for a number of cell subculture levels. The earlier subculture levels readily produced tumours but transplantability was partially reduced by the 98th serial subculture. The loss of transplantability was not closely associated with any of the other cell characteristics examined.

We wish to thank Professor C. H. Stuart-Harris for helpful discussions during the course of the study. This study was carried out with the financial assistance of the British Empire Cancer Campaign for Research.

### REFERENCES

BERMAN, L. D. AND ROWE, W. P.—(1965) *J. exp. Med.*, **121**, 955.
BERTANI, G. (1953) *Annls Inst. Pasteur, Paris*, **84**, 273.
BRADSTREET, C. M. P. AND TAYLOR, C. E. D.—(1962) *Mon. Bull. Minist. Hlth*, **21**, 96.
DARBYSHIRE, J. H.—(1966) *Nature, Lond.*, **211**, 102.
FREEMAN, A. E., CALISHER, C. H., PRICE, P. J., TURNER, H. C. AND HUEBNER, R. J.—(1966) *Proc. Soc. exp. Biol. Med.*, **122**, 835.
HUEBNER, R. J., PEREIRA, H. G., ALLISON, A. C., HOLLINSHEAD, A. C. AND TURNER, H. C.—(1964) *Proc. natn. Acad. Sci.*, **51**, 432.
HUEBNER, R. J.—(1966) ' Perspectives in Virology ', Volume **5**, ed. M. Pollard. New York (Academic Press, Inc.).
HULL, R. N., JOHNSON, I. S., CULBERTSON, C. G., REIMER, C. B. AND WRIGHT, H. F.—(1965) *Science, N.Y.*, **150**, 1044.
KITAMURA, I., VAN HOOSIER, G. JR., SAMPER, L., TAYLOR, G. AND TRENTIN, J. J.—(1964) *Proc. Soc. exp. Biol. Med.*, **116**, 563.
LANDAU, B. J., LARSON, V. M., DEVERS, G. A. AND HILLEMAN, M. R.—(1966) *Proc. Soc. exp. Biol. Med.*, **122**, 1174.
LARSON, V. M., GOSNELL, P. A. AND HILLEMAN, M. R.—(1966) *Proc. Soc. exp. Biol. Med.*, **122**, 1182.
LEVINTHAL, J. D. AND SHEIN, H. M.—(1963) *Proc. Soc. exp. Biol. Med.*, **112**, 405.
MARTI, A., CONNOR, D. J. AND SIGEL, M. M.—(1968) *J. natn. Cancer Inst.*, **40**, 243.
OXFORD, J. S. AND SCHILD, G. C.—(1967) *Br. J. exp. Path.*, **48**, 235.
POPE, J. H. AND ROWE, W. P.—(1964) *J. exp. Med.*, **120**, 577.
PULVERTAFT, R. J. V.—(1964) *Lancet*, i, 238.
ROUSE, H. C., STROHL, W. C. AND SCHLESSINGER, R. W.—(1966) *Virology*, **28**, 633.
ROWE, W. P.—(1965) *Proc. natn. Acad. Sci., U.S.A.*, **54**, 711.
SABIN, A. B. AND KOCH, M. A.—(1963) *Proc. natn. Acad. Sci., U.S.A.*, **50**, 407.
SARMA, P. S., HUEBNER, R. J. AND LANE, W. T.—(1965) *Science, N.Y.*, **149**, 1108.
SCHILD, G. C. AND SUTTON, R. N. P.—(1965) *Br. J. exp. Path.*, **46**, 263.
SMITH, K. O. AND MELNICK, J. L.—(1964) *Science, N.Y.*, **143**, 1190.
STROHL, W. A., ROUSE, H. C. AND SCHLESSINGER, R. W.—(1966) *Virology*, **28**, 645.
TRENTIN, J. J., YABE, Y. AND TAYLOR, G.—(1962) *Science, N.Y.*, **137**, 835.